# Kinetics and mineralization fraction of organic matter from sewage sludge mixed with soil under controlled laboratory conditions

**DOI:** 10.1038/s41598-022-25121-5

**Published:** 2022-12-27

**Authors:** Diogo André Pinheiro da Silva, Mateus Pimentel Matos, Marcus Vinícius Araújo Marques, Antonio Teixeira de Matos, Thiago de Alencar Neves

**Affiliations:** 1 Department of Sanitary and Environmental Engineering, Federal University of Minas Gerais (Universidade Federal de Minas Gerais), Belo Horizonte, Minas Gerais, Brazil; 2 Department of Environmental Engineering, Federal University of Lavras (Universidade Federal de Lavras), Lavras, Minas Gerais Brazil

**Keywords:** Pollution remediation, Carbon cycle

## Abstract

The objective of this work was to evaluate the kinetics and mineralization fraction of organic matter from sewage sludge mixed with the soil under controlled laboratory conditions. For this, organic material samples accumulated in layers 0–5 cm, 5–10 cm and 10–15 cm in relation to the surface of a vertical flow constructed wetland system (VF-CW) used for treatment of septic tank sludge, in addition to samples of dewatered sludge from a septic tank and a UASB reactor and non-dewatered sludge from an anaerobic digester were mixed with material collected from the surface layer of a Red Yellow Argisol at rates equivalent to the applications, via organic residues, of 300 and 600 kg ha^−1^ year^−1^ of total nitrogen. It was found that the two-phase kinetic equation fit better to the mineralization data of labile and recalcitrant organic carbon. For the lowest nitrogen application rate in the mixtures, the mineralization fractions of the total organic carbon were higher than 73%, while at the highest dose there was a reduction in the mineralization of organic matter by 17% and 63%, respectively in samples collected in layer 10–15 cm from the VF-CW and in the septic tank sludge dewatered in the drying bed.

## Introduction

The use of sewage sludge as organic fertilizer in agricultural areas has been encouraged in several countries because it can provide benefits to the soil. This organic residue contributes to an increase in the content of nutrients and organic matter in the soil, having positive effects on the physical, chemical and biological properties of this medium^1^. Furthermore, when considering the growing demand for inputs resulting from the increase in world population, sustainable nutrient sources must be made available for maintaining agricultural productivity^2^, while presenting a low environmental impact^3^.

Sewage sludge has been shown to be an excellent nutrient carrier in soil–plant systems, studies indicate that the increase in the application of sludge to the soil will proportionally increase nutrient absorption and agricultural productivity. The macronutrient contents of sewage sludge can reach 74.5, 12.5 and 1.9 g kg^−1^ DM of Nitrogen (N), Phosphorus (P) and Potassium (K), respectively^4^.

From an agronomic and environmental standpoint, encouraging the use of sewage sludge in agriculture is based on two aspects: increasing the content of organic matter in the soil and releasing mineral nutrients for the crop demand^5^. The success of this process depends on the mineralization dynamics of organic carbon applied via organic residue to the soil. Among the organic residues that can be used as a source of organic matter and nutrients for the soil, highlighted is the organic residue accumulated on the surface of vertical flow constructed wetlands (VF-CW). However, little information is available on the mineralization of these residues in the soil^6^. The prediction of organic carbon mineralization in the soil is essential for planning and implementing practices that maximize the use of organic residues in the soil^7^.

Although sanitary sewage sludge provides a large quantity of macro and micro nutrients to the soil, its application can also cause sanitary and environmental problems, therefore regulations for the use of this residue have been established in several countries. In Brazil, it is regulated by Conama Resolution No. 375/2006^8^, in which guidelines are established for the use of sludge in agricultural areas and calculation of the application rate of this residue must be performed based on its available nitrogen content. Among the variables considered in this calculation is the fraction of organic material mineralization. However, the mineralization fraction values presented in the resolution have been questioned in some studies^9^, since they do not reflect the mineralization capacity of organic material in field conditions for countries with tropical climates.

The organic matter degradation process occurs in two stages: in the first, which is intense and short, the most labile carbon compounds are exhausted and in the second stage, which is less intense and more recalcitrant, there occurs the oxidation of 65% of total organic carbon remaining in the organic residue incorporated into the soil^10^. The dynamics of organic matter mineralization from organic residues applied to soils has commonly been carried out via respirometric tests under controlled laboratory conditions^11^. These can be monitored over time, and with the obtained data it is possible to adjust mathematical models such as the first-order chemical kinetics equation ^12^ and the two-phase exponential model^13^. Two-phase model studies are used to explain the behavior of label carbon (1st phase) and recalcitrant carbon (2nd phase) mineralization^7^.

Little knowledge is available regarding the degradability potential and the mineralization fraction of organic material produced in individual or collective sanitary sewage treatment systems, and the objective of the present study was to evaluate the dynamic behavior and the mineralization kinetics of the organic material incorporated into the soil over the incubation time. This allowed for predicting its mineralization fraction and then to obtain a more real estimate of this variable when the organic residues are applied to the soil, under controlled laboratory conditions.

## Materials and methods

The organic material used in the experiment was collected from the Center for Research and Training in Sanitation of the Federal University of Minas Gerais (CePTS-UFMG), located at the Wastewater Treatment Plant of the Arrudas River Basin (WWTP-Arrudas), in the municipality of Belo Horizonte, state of Minas Gerais, Brazil, at the geographical coordinates: 19º53′42″ S and 43º52′42″ W, and elevation of 852 m.

One of the residues analyzed was collected in the surface layer, 0–15 cm thick, from a vertical flow constructed wetland system (VF-CW), after operating for 3 years with sludge from a septic tank and planted with tifton 85 grass (*Cynodon Dactylon Pers*). This surface layer was divided into 3 sublayers measuring 0–5, 5–10 and 10–15 cm deep, with collections made at random points distributed throughout the bed and homogenized to obtain a composite sample. In this text the referred layers will be represented by the respective acronyms: CWS 0–5, CWS 5–10 and CWS 10–15 cm. The other residues analyzed were septic tank sludge dewatered in a drying bed (STS), UASB reactor sludge dewatered in a drying bed (URS) and anaerobic digester sludge that was not dewatered in a drying bed (ADS). Samples in the 0–20 cm deep layer of a Ferralsols^14^, present in the UFMG experimental area, were collected to be mixed with organic residues in the present experiment.

To characterize the collected organic materials and the receiving soil, analyses were performed to determine the pH, measured in a solution prepared with 0.01 mol L^−1^ calcium chloride; easily oxidizable carbon (OOC), quantified by the Walkley–Black method; total nitrogen (TN), quantified using the Kjeldahl method; water content (WC), quantified after oven drying at 65 °C; Total solids (TS) were quantified after oven drying at 105 °C, and total volatile solids (TVS) quantified after carbonization of dry matter in the muffle furnace at 550 °C^15^. Total organic carbon (TOC) was quantified using the Total Carbon Analyzer (TOC-VCPN). Additional analyses were performed on the soil, including: phosphorus and potassium which were extracted with a Mehlich 1 solution and quantified by the spectrophotometer method; Ca^2+^, Mg^2+^ and Al^3+^ were extracted with a 1 mol KCl L^−1^ solution; potential acidity (H + Al) was extracted with a 0.5 mol L^−1^ calcium acetate solution and quantified by titration methods, and the soil granulometric composition was quantified using the pipette method^15^.

Table 1 presents the principal chemical and physical characteristics of the organic residues and the soil. Based on the results, it was verified that the soil to which the sludge was incorporated is clayey, which from an agricultural perspective presents low available phosphorus and very low exchangeable aluminum, slightly acidic pH, Ca + Mg concentrations and potential acidity; and high available potassium. Therefore, chemically there appears to be no restrictions on the activity of organic material decomposing microorganisms.

**Table 1:** Chemical and physical characteristics of the soil (air dried fine earth) and the organic materials collected at the depths of 0–5 cm (CWS 0–5), 5–10 cm (CWS 5–10) and 10–15 cm (CWS 10–15) of the VF-CW as well as septic tank sludge dewatered in a drying bed (STS), UASB reactor sludge dewatered in a drying bed (URS) and anaerobic digester sludge that was not dewatered in a drying bed (ADS).

Soil			Organic residues							
Variables	Units	Values	Variables	Units	CWL 0–5	CWL 5–10	CWL 10–15	STS	URS	ADS
pH**	–	4.61	pH**	–	5.2	5.8	5.5	5.3	5.9	7.4
OOC*	(dag kg^−1^)	1.23	OOC *	(dag kg^−1^)	15.75	10.05	9.45	36.00	23.55	27.30
TOC*	(dag kg^−1^)	1.60	TOC *	(dag kg^−1^)	27.84	18.33	15.99	62.91	43.13	48.45
TN*	(dag kg^−1^)	0.13	OOC/TOC	–	0.57	0.55	0.59	0.57	0.55	0.56
K^+^*	(mg dm^−3^)	141.0	TN*	(dag kg^−1^)	2.17	1.26	1.24	3.40	3.67	5.24
Ca^2+^*	(cmol_c_ dm^−3^)	2.29	C/N	–	9.4	10.4	9.9	13.8	8.3	6.8
P_disp_*	(mg dm^3^)	2.4	WC**	(dag kg^−1^)	37.47	36.26	39.79	32.9	39.26	97.56
Al^3+^*	(cmol_c_ dm^−3^)	0.10	TS**	(dag kg^−1^)	61.45	62.86	59.47	60.04	56.25	2.37
H + Al*	(cmol_c_ dm^−3^)	3.3	TVS**	(dag kg^−1^)	38.63	25.64	22.84	42.38	33.94	66.46
Clay*	(dag kg^−1^)	40.0								
Silt*	(dag kg^−1^)	8.3								
Sand*	(dag kg^−1^)	13.5								
WC**	(dag kg^−1^)	12.43								

*OOC* easily oxidizable carbon, *TOC* total organic carbon, *TN* total nitrogen, *K* potassium, *Ca* calcium, *P* phosphorus, *Al* aluminum, *H* + *Al* potential acidity, *WC* water content, *TS* total solids, *TVS* total volatile solids.

*Quantified on a dry basis.

**Quantified on a wet basis.

To assess carbon mineralization, aerobic incubation of the residue-soil and control soil samples was carried out in hermetic containers (respirometers) at a temperature of ± 26 °C and a water content of around 70%, according to the respirometric method of Bartha^11^. This process is based on the capture of carbon dioxide released by microbial activity during the organic material decomposition process in a standardized sodium hydroxide solution (NaOH, 0.25 mol L^−1^). Estimates of the amounts of released CO2 were calculated from electrical conductivity data in the NaOH solution^16^.

The doses of 300 kg ha^−1^ of N (dose I) and 600 kg ha^−1^ of N (dose II) were used for the application of organic residues to the soil, considering 100% mineralization of the organic material. Monitoring of organic carbon mineralization, at the dose of application I, was carried out on days 1, 2, 4, 6, 9, 12, 15, 18, 22, 27, 32, 37, 46, 56, 66, 76, 86, 100, 118 and 140; and for application dose II was performed at 1, 4, 8, 11, 14, 18, 23, 28, 39, 42, 52, 62, 72, 82, 96, 114 and 140 days after incorporation of the organic residue. The experiment was conducted with 100 g of the residue-soil mixture using three repetitions for dose I and two repetitions for dose II, and three repetitions with the control soil, totaling 36 respirometers. The lowest dose was equivalent to the application of residue/TOC in the respective containers of 22.68 Mg ha^−1^ of CWS 0–5; 35.79 Mg ha^−1^ of CWS 5–10; 36.48 Mg ha^−1^ of CWS 10–15; 35.42 Mg ha^−1^ of the STS; 23.15 Mg ha^−1^ of the URS and 258.44 Mg ha^−1^ from ADS. Dose II was equivalent to the application of 45.36 Mg ha^−1^ of CWS 0–5; 71.58 Mg ha^−1^ of CWS 5–10; 72.96 Mg ha^−1^ of CWS 10–15; 70.84 Mg ha^−1^ of the STS; 46.30 Mg ha^−1^ of the URS and 516.88 Mg ha^−1^ of the ADS.

To describe C mineralization in the studies sampled two mathematical models were used. The simple exponential first order kinetic model—Eq. (1)^12^, and the two-phase kinetic model—Eq. (2)^13^.

1 $${{\varvec{C}}}_{{\varvec{t}}} = {{\varvec{C}}}_{0}. \left(1-{\varvec{e}}^{-{\varvec{k}}.{\varvec{t}}} \right),$$

2 $${{\varvec{C}}}_{{\varvec{t}}} = {{\varvec{C}}}_{1}. \left(1-{\varvec{e}}^{-{\varvec{k}}1.{\varvec{t}}} \right)+ {{\varvec{C}}}_{2}. \left(1-{\varvec{e}}^{-{\varvec{k}}2.{\varvec{t}}} \right),$$

where ${{\varvec{C}}}_{{\varvec{t}}}$ is the accumulated quantity of mineralized organic carbon (mg CO_2_ /0.1 kg of soil-residue) at time t (days), ${{\varvec{C}}}_{0}$ represents the amount of potentially mineralizable C (mg CO_2_ /0.1 kg of soil-residue) and ${\varvec{k}}$ is the mineralization rate constant (day^-1^). ${{\varvec{C}}}_{1}$ and ${{\varvec{C}}}_{2}$ respectively represent the labile and recalcitrant fractions of C mineralization at the specific rates of ${{\varvec{k}}}_{1}$ and ${{\varvec{k}}}_{2}$. The sum of ${{\varvec{C}}}_{1}$ and ${{\varvec{C}}}_{2}$ has the same physical meaning as ${{\varvec{C}}}_{0}$ in the exponential first order kinetics model.

The parameters of the adjusted models for each evaluated treatment were calculated by non-linear regression analysis, using the Sigma Plot 13.0 program from the accumulated C-CO_2_ data. The determination coefficient (R^2^) was used to compare which model fit best.

The mineralization fraction of the accumulated organic C was calculated in a fashion similarly to that conducted by Garcia et al*.* (1992). The net mineralization fraction (NMF) and complementary mineralization fraction (CMF) were calculated according to Eqs. (3) and (4), based on the accumulated amount of C-CO_2_ released in the 140 day period.

3 $${\varvec{N}}{\varvec{M}}{\varvec{F}} = {({\varvec{C}}}_{{\varvec{A}} }/{({\varvec{C}}}_{{\varvec{C}}}+{{\varvec{C}}}_{{\varvec{B}}})).100,$$

4 $${{\varvec{C}}{\varvec{M}}{\varvec{F}} = (({\varvec{C}}}_{{\varvec{A}} }- {{\varvec{C}}}_{{\varvec{D}}})/{{\varvec{C}}}_{{\varvec{C}}}).100,$$

where ${{\varvec{C}}}_{{\varvec{A}}}$ is the total accumulated C released as CO_2_, ${{\varvec{C}}}_{{\varvec{B}}}$ represents the total content of organic C in the soil, ${{\varvec{C}}}_{{\varvec{C}}}$ is the total content of organic C added with the organic residue and ${{\varvec{C}}}_{{\varvec{D}}}$ is the total accumulated C released from the control soil in the form of CO_2_.

The mineralization fraction of the potentially mineralizable accumulated organic C, estimated using Eq. (1)^17^ and Eq. (2)^9^. The potentially mineralizable fractions (${{\varvec{M}}{\varvec{F}}}_{{\varvec{C}}{\varvec{A}}{\varvec{L}}1}$ and ${{\varvec{M}}{\varvec{F}}}_{{\varvec{C}}{\varvec{A}}{\varvec{L}}2}$) were calculated as described in Eqs. (5) and (6).

5 $${{{\varvec{M}}{\varvec{F}}}_{{\varvec{C}}{\varvec{A}}{\varvec{L}}1}=(({\varvec{C}}}_{0({\varvec{C}}{\varvec{A}}{\varvec{L}}1)}-{{\varvec{C}}}_{0\left({\varvec{C}}{\varvec{D}}\right)})/{ {\varvec{C}}}_{{\varvec{C}}}).100,$$

6 $${{\varvec{M}}{\varvec{F}}}_{{\varvec{C}}{\varvec{A}}{\varvec{L}}2}={(({\varvec{C}}}_{0({\varvec{C}}{\varvec{A}}{\varvec{L}}1)}-{{\varvec{C}}}_{0\left({\varvec{C}}{\varvec{D}}\right)})/{ {\varvec{C}}}_{0}).100,$$

where ${{\varvec{C}}}_{0({\varvec{C}}{\varvec{A}}{\varvec{L}}1)}$ is the total potentially mineralizable C–CO_2_ estimated and recalculated for the time of 140 days, ${{\varvec{C}}}_{0({\varvec{C}}{\varvec{D}})}$ is the total potentially mineralizable C–CO_2_ in the control soil, ${{\varvec{C}}}_{{\varvec{C}}}$ is the total organic C added with the organic residue and ${{\varvec{C}}}_{0}$ is the total estimated potentially mineralizable C–CO_2_.

The analytical data was subjected to the ANOVA test, and comparison of the means was performed using the Tukey test at a significance level of 5%.

## Results and discussion

**Figure 1:**
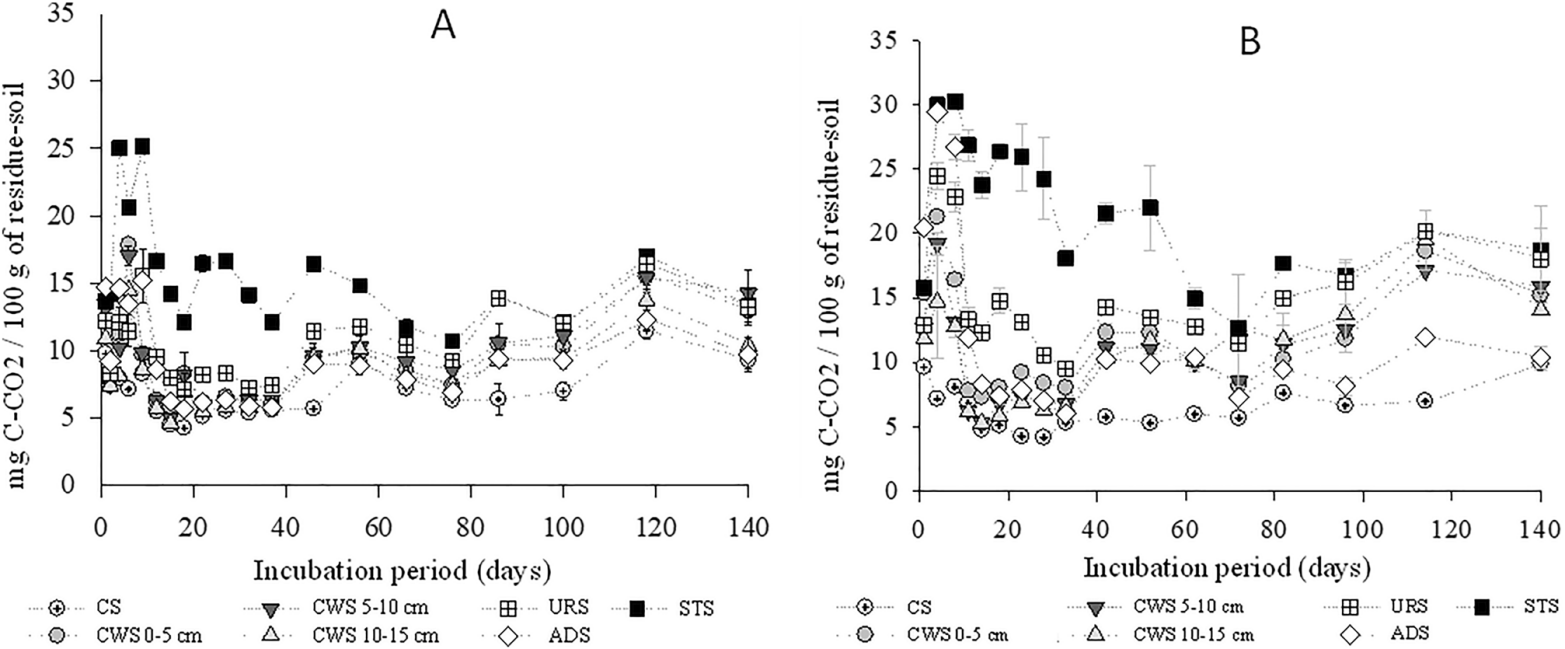
Quantity of carbon released in the form of CO_2_ from the soil that was incorporated with the organic residue in dose I of 300 kg ha^−1^ of N (**A**) and in dose II of 600 kg ha^−1^ of N (**B**) and in the control soil, during the 140-day incubation period.

As can be observed in Fig. 1, during the initial period of the experiment the daily mineralized quantity of CO_2_ was greater in the residue-soil mixture to which the STS was incorporated than in the mixtures where the other organic residues were incorporated. This is due to the higher TOC content in the STS, which is 62 dag kg^-1^, while in the other organic residues TOC is between 15 and 49 dag kg^−1^ according to values shown in Table 1. Research reported that sewage sludge stabilized in aerobic and anaerobic treatment systems has a smaller labile fraction, and therefore slower mineralization of the organic carbon^18^.

Annual crops normally consume an average of 100 kg ha^−1^ of N, having at least 3 harvests per year, that is, they consume an average of 300 kg ha^−1^ yr^−1^^10^. Studies show that the mineralization of organic matter can be low depending on the climatic conditions of the place, therefore, when sewage sludge is applied in order to meet the nutritional demands of the crops, higher doses must be applied and can reach double, for this reason, this study tested the dose of 600 kg ha^−1^.

In all treatments the maximum mineralization of organic carbon occurred in the first 20 days of incubation, both when applying dose I and dose II (Fig. 1). Research observed that the mineralization of organic carbon, nitrogen and phosphorus from shrub residues, in semi-arid soil, occurred in greater intensity in the first month, a period when components of easier degradation are available^19^.

**Figure 2:**
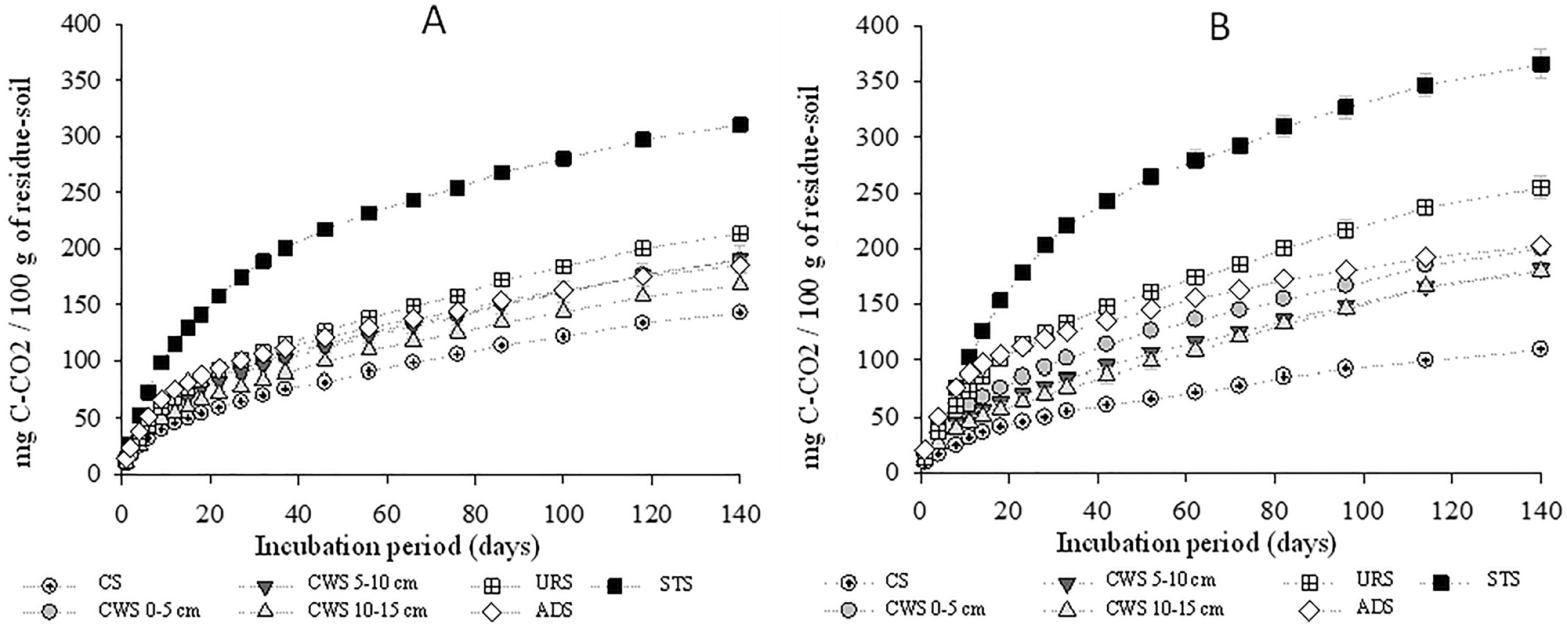
Accumulated quantity of carbon released in the form of CO_2_ from soil that was incorporated with the organic residue in dose I of 300 kg ha^−1^ of N (**A**) and in Dose II of 600 kg ha^−1^ of N (**B**) and the control soil, during the 140-day incubation period.

As shown in Fig. 2, the accumulated values of C released in the 140 day period were higher in dose II (Fig. 2B) than in dose I (Fig. 2A) for soils mixed with the organic residues. In study on the decomposition of sewage sludge and sewage sludge compost in Halic Alic Nitosol (Hapludox) soil, clay texture, observed an increase in the evolution of C–CO_2_ when the sludge dose applied to the soil was increased^20^. The accumulated mineralized C from VF-CW residues in its mixture with the soil decreased with the increasing depth of its collection, i.e., it was proportional to the time that the residue remained in the system. Research reported a reduction in the TOC content present in sludge collected in the vertical profile of the CW, which were 18.1, 15.2 and 14.6% in the 0–40, 40–80 and 80–110 cm layers, respectively^21^. Also found greater stability of the organic fraction of organic residue accumulated in the lower layers of a VF-CW^22^.

**Figure 3:**
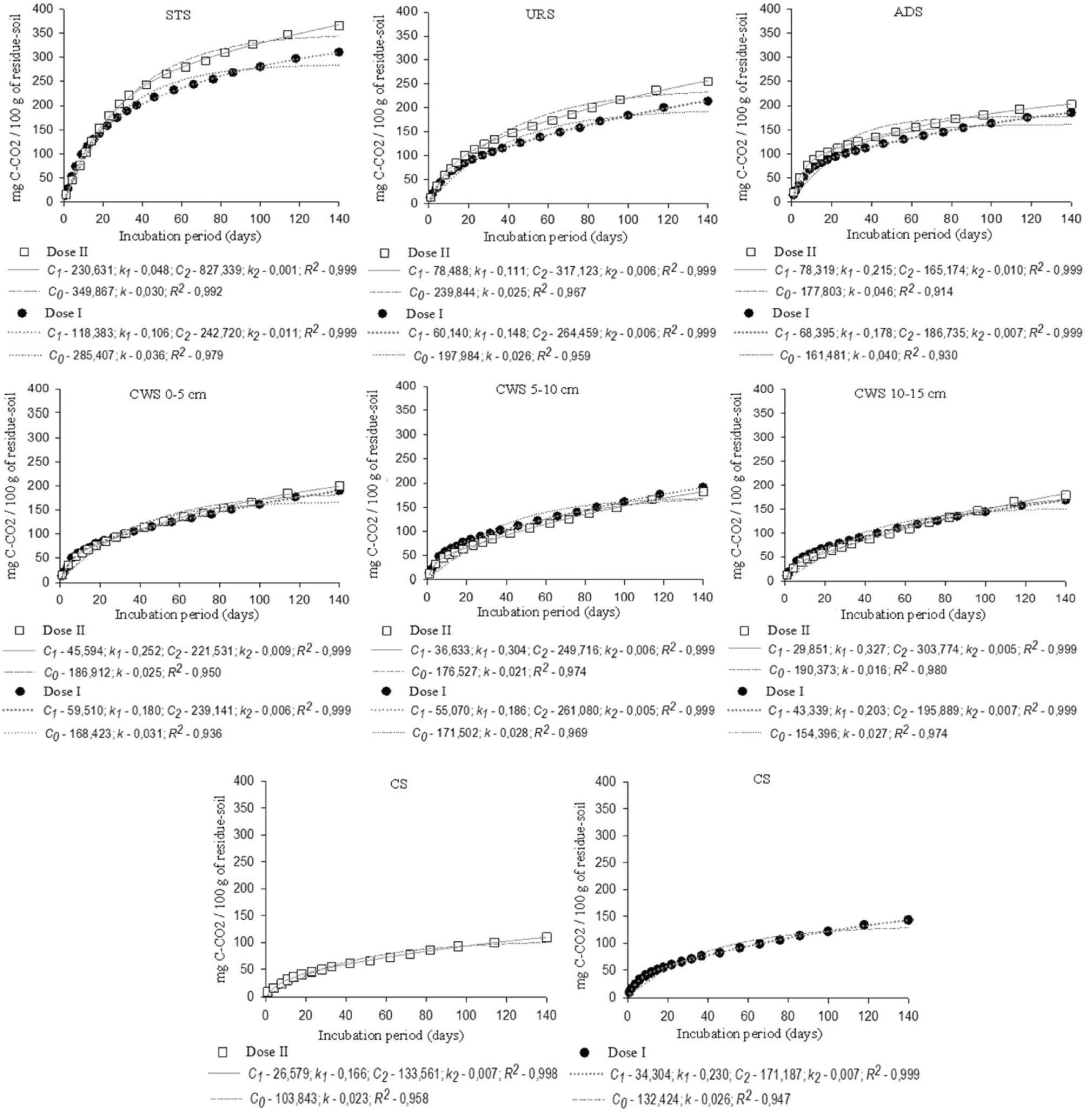
Determination coefficient (R2) and model parameters adjusted to the accumulated CO_2_ values in the 140-day period, in doses I and II of 300 kg ha^−1^ of N and 600 kg ha^−1^ of N, respectively.

Figure 3 shows the parameters and coefficients of determination (R^2^) used to compare the models adjusted to the accumulated C–CO_2_ release data from the treatments. The coefficients adjusted well to the data at a significance level of 5%, corroborating with observations similar to those obtained in another research^23^. The models provided an adequate description of the mineralization kinetics of organic carbon in all samples, especially the two-phase kinetic model which presented R^2^ very close to 1, in agreement with the studies in an arid soil^24^. The first-order kinetic model also adequately described the behavior of organic carbon mineralization in the initial phase, however the two-phase kinetic model described the kinetics of both labile, easily degradable compounds present at the beginning of the process, as well as recalcitrant compounds in the second phase.

The labile C values (*C*_*1*_), in the two-phase model for the two application doses, were higher in the mixture of STS with soil than in those of URS, ADS and VF-CWS with soil. When assessing mineralization (*C*_*1*_) within doses I and II, there was an increase in *C*_*1*_ when increasing the dose in mixtures of STS, URS and ADS with soil and the opposite behavior for mixing VF-CWS with soil, in which *C*_*1*_ presented higher values when dose I was applied. According with studies, the incorporation of high organic matter doses to the soil can contribute to overcome the microbial capacity of the medium and effect its degradation, which can reduce the mineralization rate^25^. The labile fraction (*C*_*1*_) represents, in mixtures of CWS 0–5, CWS 5–10 and CWS 10–15 cm with soil, between 8 and 20%, and in the CS 16% of the total potentially mineralizable organic carbon (*C*_*1*_ + *C*_*2*_). The proximity of these values to those obtained in the CS suggests that the organic matter contained in the organic residue accumulated in the VF-CWS is highly stable.

The *k*_*1*_ constants adjusted for the first phase reactions in more labile compounds, in the two organic residue application doses, were higher in the residue-soil mixtures and control soil, however, the VF-CWS showed values close to that of the control soil. Similarly to what was verified in this experiment, observed that after successive applications of sewage sludge to the soil, greater mineralization occurred in the initial 28 days after incubation of the residue^26^. In the present study, the high mineralization coefficients (*k*) obtained in the first days can be justified by the relatively high OOC/TOC ratio of the organic residue, which was 0.57 as shown in Table 1. Research evaluated mineralization of sewage sludge compost (181 g kg^-1^ of TOC) and thermally dried sludge (296 g kg^−1^ of TOC), with respective *k*_*1*_ values of 0.024 and 0.046, lower than those observed in the present study^24^.

Recalcitrant C (*C*_*2*_), in the two organic residue application doses, was higher than 80% in the mixtures of URS, CWS 0–5, CWS 5–10 and CWS 10–15 with soil and the CS, indicating that the organic matter of these residues is slowly mineralized. Slightly lower percentages (between 67 and 80%) were obtained in the mixtures with STS and ADS. Study observed that the behavior of organic compound mineralization takes place in two phases: the first, from 0 to 14 days is considered fast, and the second, after the 14th day is considered slow^7^. The adjusted mineralization constants *k*_*2*_ are similar to those observed by another study, who obtained values between 0.007 and 0.015 for *k*_*2*_ when incorporating VF-CWS to the soil under field conditions^10^.

The net mineralization fractions (MF_NMF_), in dose I and dose II, after 140 days of sample incubation were less than 19% for all residue-soil mixtures and also in the control soil, with the highest values found in the STS-soil mixture (Table 2). Data on the complementary mineralization fraction (MF_CMF_), in the two application doses, were lower in the FV-CWS-soil mixtures, together with that of the URS-soil mixture, and higher in the STS-soil mixture. The MF_NMF_ values obtained in this work are lower than those obtained in other studies, which cite values in the range of 20 to 60% of the total C added after being mixed with the soil, for the incubation time between 100 and 130 days^27^.

**Table 2:** Net mineralization fraction (NMF), complementary mineralization fraction (CMF) and estimated mineralization fraction (MF_CAL1_, MF_CAL2_) obtained from the adjusted parameters in the two simultaneous phase kinetic equation, for data of the organic residues and control soil at the doses of 300 kg ha^-1^ of N and 600 kg ha^-1^ of N, monitored during the 140 day period.

Organic residue	MF_NMF_	MF_CMF_	C_pot_	C_pot(recal1)_	MF_cal1_	MF_cal2_	C_pot(recal2)_	MF_cal3_
	%		mg C–CO_2_		%		mg C–CO_2_	%
	140 days						365 days	
**Dose I (300 kg ha**^**−1**^** of N)**								
CWS 0–5 cm	11.53b	19.00ab	298.651	195.141	21.80	57.86	271.887	85.55
CWS 5–10 cm	11.61bc	19.62ab	316.150	186.502	18.62	40.90	274.059	73.98
CWS 10–15 cm	10.45ab	12.07a	239.228	165.709	11.99	72.52	224.009	94.31
STS	18.71c	64.76c	361.103	309.068	64.94	100	356.724	100
URS	12.10b	19.34ab	324.599	210.316	18.95	58.00	295.002	86.32
ADS	11.90b	27.22b	255.13	185.046	28.46	88.24	240.622	97.57
CS	9.27a	–	205.491	141.243	–	–	192.191	–
**Dose II (600 kg ha**^**−1**^** of N)**								
CWS 0–5 cm	10.95bc	20.97a	267.125	204.287	21.75	88.12	258.831	100
CWS 5–10 cm	9.85b	16.21a	286.349	178.544	15.30	54.30	258.402	86.08
CWS 10–15 cm	10.28b	20.19a	333.625	182.775	20.94	41.94	284.651	77.75
STS	18.79d	49.23c	1057.970	338.438	41.75	25.44	483.634	37.19
URS	12.54c	22.96ab	395.611	258.706	23.45	63.15	360.120	89.33
ADS	11.86bc	30.04b	243.493	202.762	29.88	100	239.030	100
CS	7.10a	–	160.140	110.013	–	–	149.763	–

MF_(cal3)_, calculated in accordance with MF_(cal2) but_ for the period of 365 days.

The mineralization fractions MF_cal1_ and MF_cal2_, calculated using the adjusted parameters for the two-phase kinetic equation, were estimated for 140 and 365 days. Based on the data presented for MF_cal1_, in dose I the values were similar to those reported for MF_CMF_. The MF_cal2_ presented values between 40 and 73% in the mixtures of FV-CWS and URS residues in the soil, presenting higher percentages for the mixtures of ADS and STS in the soil of 88 and 100%, respectively, i.e., there is greater mineralization of organic matter from non-stabilized residues. Similar ranges, between 20 and 60%, were observed that the mineralization of organic carbon added via biosolids after a 130 day incubation period^28^.

The MF_cal3_ estimates, at 365 days of incubation of the residue-soil mixtures for dose I, values greater than 73% for all evaluated organic residues. Alkalized secondary sludge in Haplic Cambisol Tb latosol dystrophic, at a dose of 500 kg ha^−1^ of N under field conditions, presented annual mineralization of 100%^17^. In dose II there was a reduction in the mineralized percentage in relation to that obtained when dose I was applied. In the mixture of CWS 10–15 with the soil, in the dose of 72.96 Mg ha^−1^, there was a 17% reduction, and for the mixture with STS in the dose of 70.84 Mg ha^−1^, there was a reduction of 63%. The application of anaerobically digested sludge to Red Latosol, in doses higher than those recommended for the cultivation of corn, affected the microbiota responsible for carbon mineralization in the soil, as well as its chemical characteristics^29^. Study found that microbial activity increased with the application of sludge from an industrial landfill sewage treatment plant when applied to Dystrophic Red-Yellow Argisol in doses of 0, 2, 10, 25 and 50 Mg ha^−1^, with carbon mineralization being proportional to the added doses^30^.

Also based on the results obtained it is possible to verify the rapid stabilization of the organic material obtained from the VF-CW and the potential for its use in agricultural areas, due to its high organic matter content and potential as a fertilizer. The URS, ADS and STS showed higher quantities of mineralizable organic matter, and can also be used for agricultural purposes provided that special care is taken for handling, monitoring the dose to be applied and disposal of these organic residues in the soil.

## Conclusion

The organic residues from a VF-CW showed greater biochemical stability than the sludge from anaerobic processes in WWTPs, and this stability increased in the deeper layers. The mineralized organic residue fractions in the 140 day period, under laboratory conditions (water content 70% and soil temperature 28 °C) were higher than 40% and exceeded 73% in the 365 day period, for the application dose of 300 kg ha^−1^ of N. When applying the N dose of 600 kg ha^−1^ there was a reduction in the fraction of organic matter mineralization to 17% and 63%, respectively in mixtures of CWS 10–15 cm with soil and STS with soil. The two-phase kinetic equation was that which best fit to the mineralization data of labile and recalcitrant organic carbon in the organic residue-soil mixtures. The highest organic matter mineralization rates occurred in the first 20 days of sample incubation, a period in which 20 to 35% of the quantified organic carbon was degraded. The mineralization of residues was above 73%, for a dose of 300 kg ha^−1^ yr^−1^, making available for agriculture an amount of nitrogen of 219 kg ha^−1^ yr^−1^. The mineralization of the residues was above 77%, for a dose of 600 kg ha^−1^ yr^−1^, making available for agriculture an amount of nitrogen of 462 kg ha^−1^ yr^−1^, with the exception of the STS residue.

## Data Availability

All data generated or analyzed during this study are included in this published article in the form of figures, tables and graphs.
